# Available techniques to minimize scars in surgical management of gynecomastia – a comprehensive review

**DOI:** 10.1016/j.jpra.2024.09.011

**Published:** 2024-09-18

**Authors:** G. Frigerio, A. Serre, P.E. Engels, D.F. Kalbermatten, D. André-Lévigne

**Affiliations:** a Division of plastic and reconstructive surgery, University Hospital of Geneva, Geneva, Switzerland; b Regenerative medicine and reconstructive surgery research group, Department of Surgery, University of Geneva, Geneva, Switzerland

**Keywords:** Gynecomastia, Male chest, Liposuction, Hemostatic net, Skin excess, Hematoma prevention, Aesthetic surgery

## Abstract

**Objective:**

Gynecomastia and lipomastia are benign proliferations of the male breast affecting 32–65% of men. Numerous surgical procedures often result in stigmatizing scars when it comes to skin resection. The purpose of this study was to review skin-sparing techniques and to describe our skin-sparing approach to treat skin excess using transcutaneous netting.

**Materials and Methods:**

A comprehensive review of the literature was conducted aiming at identifying available techniques to avoid skin resection in gynecomastia or lipomastia patients (Simon's grade IIb and III). Surgical techniques, patient satisfaction, time of follow-up, and complications were assessed.

**Results:**

Seven studies detailed skin retraction techniques, including laser-, ultrasound-, and radiofrequency-assisted liposuction (LAL, UAL, and RAL), microneedling, and nipple-areolar complex (NAC) plaster lifting. All articles provided Simon's grade classification, with most studies including patients with and without skin laxity. Complication rates were low (1.5–10%), and patient satisfaction ranged from 87.5% to 100%.

While transcutaneous netting has been reported to reduce hematoma in gynecomastia surgery, no studies specifically examined its role in managing skin redundancy.

**Conclusions:**

Limited data exist on scarless skin retraction techniques for gynecomastia. While LAL, UAL, and RAL show some potential, controlled studies are lacking, and skin resection is often performed for high skin redundancy. We recommend a skin-sparing approach using liposuction and transcutaneous netting for gynecomastia up to Simon's grade III, which allows for skin retraction and NAC fixation. No literature was found assessing the efficacy of transcutaneous netting in promoting skin retraction in gynecomastia.

## Introduction

Plastic surgeons are frequently confronted with the surgical management of benign enlargement of the male breast, gynecomastia or lipomastia, as it has been reported to affect 32–65% of all men, of which a significant proportion desire surgical correction.^1^ Gynecomastia is a benign proliferation of the glandular tissue of the male breast, whereas lipomastia is due to a diffuse increase of fat tissue.^2^ In adult patients, the etiology may vary but the most common cause remains idiopathic.^3^

Many of these patients show poor self-image, depression, anxiety, and social phobia.^4^^,^^5^ Patient-reported outcome after definitive surgical correction has been shown to have a clear positive impact, especially on physical and psychological wellbeing.^6^ Overall patient satisfaction after surgical correction has been reported with an average of 84.5%.^7^

The degree of gynecomastia is often characterized based on a qualitative assessment of skin redundancy and breast volume such as Simon's classification where starting from grade IIb surgeons are confronted with a skin excess^8^ (Table 1). Accordingly, lipomastia can be surgically assessed quantifying fat tissue excess and skin redundancy.

**Table 1:** Simon's classification.

Grade	Breast enlargement	Skin excess
I	Small	No
IIa	Moderate	No
IIb	Moderate	Yes
III	Marked	Yes

Simon's grade IIb and III are classically considered to be candidates for skin resection.

Multiple surgical approaches for the correction of gynecomastia and lipomastia have been described, including minimally invasive techniques such as liposuction, vacuum-assisted mastectomy, and endoscopic mastectomy (pull-through techniques); surgical glandular resection is commonly done utilizing a hemiareolar incision.^9^ If significant skin redundancy is present, most authors still perform a skin resection, which can be done in a variety of patterns. The eccentric skin resection with a purse string allows for nipple-areolar complex (NAC) reshaping and repositioning at the same time; however, it can be used only in case of limited skin excess and is associated with high complication rates.^10^ In cases of abundant skin excess, the customary procedure is transverse mastectomy with free nipple grafting, which not only leaves an extensive, visible scar and potentially discoloration of the NAC but also accentuates the inframammary fold (IMF), giving a more feminine appearance to the breast.^9^^,^^10^ Another option involves the “hockey stick” skin excision, which tightens the skin laterally with a scar lying at the lateral pectoral border. The disadvantage of this technique is the possible lateralization of the NAC and some expected residual skin excess. The boomerang pattern corrects the nipple ptosis and the anterolateral skin excess by leaving a long scar visually interrupted by the areola. This technique is technically demanding and often leaves pigmented and thick scars; the risk of NAC necrosis is also present.^9^^,^^10^

Many authors also advocate a combination of previously described procedures, notably the combination of sharp glandular resection and liposuction, to homogenize results.^1^^,^^10^ An example is given by Fan et al.^11^ utilizing ultrasound-assisted liposuction (UAL) followed by an endoscopic resection.

Burger et al. investigated postoperative patients’ satisfaction concerning the appearance of the chest wall and scars, using a modified BREAST Q^Ⓡ^ questionnaire, after subcutaneous mastectomy using either periareolar or submammary incisions.^12^ They did not find any statistically significant difference between the groups, but periareolar incisions were associated with a statistically significant higher complication rate.^12^

The most common major complication for gynecomastia surgery is hematoma, with an average presentation of 5.8%.^1^ In many fields of plastic surgery, different techniques have been developed to reduce the risk of developing seroma/hematoma, one of them being internal suturing,^13^^,^^14^ also referred to as quilting, which allows for closure of the dead space created during dissection. In 2012, Auersvald et al. described a new technique based on an external hemostatic net (also called external quilting or netting) composed of a series of continuous transfixing sutures during rhytidectomy.^15^ This technique secures the skin to the underlying muscular fascia through running sutures, reducing dead space and improving hemostasis.

In fact, this procedure has grown in popularity for hemostatic purposes as described by multiple authors in rhytidectomy,^16^^,^^17^ neck lifts,^18^^,^^19^ and gynecomastia.^20^ Auersvald et al. have published a retrospective analysis of 500 patients using external quilting in rhytidectomy with impressive results.^18^

In female breast surgery, the use of netting showed that skin redundancy could be effectively reduced in secondary breast surgery, where patients refused mastopexy scars.^21^ It is advocated that netting can stabilize the IMF and help in tightening excessive skin.^21^

There have been increased concerns about dermal perfusion with external quilting; however, different studies could demonstrate the safety of this technique.^22^^,^^23^ No comparison between above mentioned scarless techniques and netting has been assessed yet.

Based on the above-mentioned elements, we advocate the use of transcutaneous netting combined with liposuction in the surgical treatment of lipomatous and glandular gynecomastia in patients up to Simon's grade III. We believe that netting may not only secure the skin and NAC in the desired position but also offer a skin-tightening effect. The various sutures may create a “microneedling effect” that promotes skin retraction. By combining its hematoma prevention with skin tightening, we find this technique suitable for most patients, except those with extreme skin excess or very poor skin quality. In addition, we believe that this technique allows for a good repositioning of the NAC and an adequate definition of the lower and lateral border of the pectoralis major muscle, giving a masculine appearance to the breast while avoiding stigmatizing scars.

The purpose for this study is to conduct a comprehensive review summarizing currently available data on scarless skin retraction techniques in gyneco- and lipomastia. We then describe our preferred technique to treat skin excess and to effectively determine the postoperative position of the NAC using transcutaneous netting sutures.

## Materials and Methods

A comprehensive review of the literature was conducted to identify articles describing techniques that induce skin retraction without the need for skin resection.

Studies included in this review needed to match the following inclusion criteria: a study includes cis-male patients with gynecomastia or pseudogynecomastia with skin excess (Simon's grade ≥ IIb) undergoing surgical interventions. Articles were included when techniques to avoid skin resection were described specifically for patients presenting skin excess.

A comprehensive search was conducted by screening the PubMed bibliographic database, EMBASE, and Cochrane using the keywords (“excess skin” OR “skin tightening” OR “body contouring” OR “non-surgical” OR “male breast skin reduction” OR “netting” OR “quilting” OR “minimal invasive” OR “ultrasound-assisted” OR “radiofrequency” OR “microneedling” OR “plaster” OR “skin-sparing”) AND (“pseudogynecomastia” OR “lipomastia” OR “gynecomastia”).

A single reviewer screened and selected relevant articles that matched the inclusion criteria of skin-sparing management of skin excess in gynecomastia and pseudogynecomastia with particular attention to skin tightening.

## Results

Our comprehensive review of the literature revealed that there is a lack of data on techniques to achieve scarless skin retraction in the management of gynecomastia.

**Figure 1:**
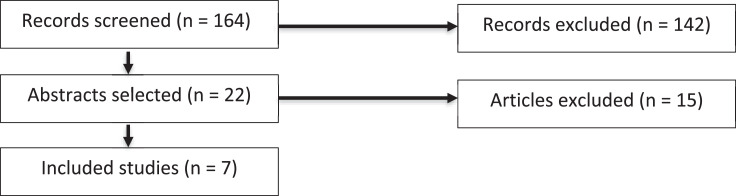
Flowchart of paper selection process for a comprehensive review. 164 records corresponded to the search criteria. 142 papers were excluded based on abstract screening for not meeting all inclusion criteria. After 22 full text reads, 7 articles were included in this review.

164 records corresponded to the initial search criteria. 142 papers were excluded based on abstract screening for not meeting all inclusion criteria. After 22 full text reads, 7 articles were included in this review (Figure 1).

Two articles were retrospective studies, one a review describing case series using their technique, the other a technique description, and the rest were case series (Table 2).

**Table 2:** Details of studies included in the review.

Author	Type of study	Year	Patients n°	Mean age (range)	BMI (range)	Simon's grade	Time of follow-up	Patient reported outcomes
Ramasamy K. et al.	Retrospective study	2023	448	26.6 (14–55)	27.31 kg/m^2^ (17.2–46.7 kg/m^2^)	Grade I: n = 21, 4.7% Grade IIa: n = 236, 52.7% Grade IIb: n = 133, 29.7% Grade III: n = 36, 8%	14 months	Overall satisfaction: 67.9% very satisfied, 25% satisfied, 7.1% neutral; Scar: 92.9% satisfied, 7.1% not satisfied; Confidence gain: 96.4% yes, 3.6% maybe; Desire of revision: 64.3% no, 28.6% maybe, 7.1% yes
Ramasamy K. et al.	Retrospective study	2022	30	-	-	Grade IIb: n = 19, 63%; Grade III: n = 11, 37%	6 months	No patient asked for revision
Hurwitz D. et al.	Review including the description of the technique	2022	1	45	-	Grade IIb	-	-
Theodorou S. et al.	Technique description	2018	312	31.4	23.2	Grade IIb	12 months	89% would recommend the procedure
Trelles M, et al.	Case series	2013	28	35.5 (24–56)	27.21 kg/m^2^ (21–36kg/m^2^)	Grade IIa: n = 1, 3.6% Grade IIb: n = 2, 7.1% Grade III: n = 25, 89.3%	6 months	Very good result: 64.3% Good: 21.4% Fair: 10.7% Poor: 3.6%
Trelles M. et al.	Case series	2013	32	38 (22–64)	26.06 kg/m^2^ (20–36.6 kg/m^2^)	Grade < IIb: n = 23, 28.1% Grade ≥ Iib: n = 9, 71.9%	6 months	Very good: 71.9%; Good: 21.9%; Fair: 6.2%
Esme D. et al.	Case series	2007	28	57 (17–80)	-	Grade I: n = 5, 17.9% Grade IIa: n = 12, 42.8% Grade IIb: n = 8, 28.6% Grade III: n = 3, 10.7%	8 weeks	Shape: good 14.3% and excellent 85.7%; Symmetry: good 14.3% and excellent 85.7%; Scar: good 3.6% and excellent 96.4%

The following techniques were described to achieve scarless skin retraction:

### Laser-assisted liposuction (LAL)

Trelles et al. described their approach to promote skin retraction in gynecomastia using LAL in two case series.^24^^,^^25^

Twenty-eight patients with gynecomastia and skin excess were enrolled: one presented Simon's grade IIa, the other grade IIb, and the remaining had grade III gynecomastia. Liposuction was conducted with a special fiber canula emitting diode laser.

During the follow-up of 6 months, the authors report clear skin retraction evaluated subjectively by the patients with a visual analogue scale and objectively by the surgeon with chest measurements. A significant difference (p < 0.001) in mean chest circumference between preoperative (117.4 ± 11.1 cm) and postoperative measures (103.3 ± 7.5 cm) was found. After 6 months, 64.3% of patients scored the results as “very good,” 21.4% as “good,” 10.7% as “fair,” and 3.6% as “poor.” Skin flaccidity improved visually until the last control at 6 months: particularly notable was the disappearance of the shadow typically observed below the low breast pole. The postoperative period was incident-free.^24^

### Ultrasound-assisted liposuction

Esme at al.^26^ published a study on 28 patients, of which 40% with moderate to severe gynecomastia (grade IIb and more) were included. After performing UAL, the residual glandular tissue was directly excised. Cosmetic results were assessed through photographs pre- and postoperatively at 2 and 8 weeks. Eight weeks postoperatively, thorax shape and symmetry were scored by 85% of patients as “excellent” and 15% as “good,” and 96% of participants reported an “excellent” outcome regarding the scar, and only 4% a “good” result. None of the measured parameters were reported as “poor.”^26^

### Radiofrequency-assisted liposuction (RAL) and microneedling

Electromagnetic radiation can be applied on tissues through RAL, such as BodyTite (InMode Corp., Toronto, CA) to reduce skin laxity and breast ptosis.^27^ Hurwitz et al. described this method for patients with grade IIb gynecomastia in a review using BodyTite followed by liposuction.^9^ Some cases, described as presenting wrinkled skin after the procedure, benefited also from microneedling via Morpheus 8 Body (InMode Corp., Toronto, CA). The anecdotal data show photographs at 1, 2, and 7 months with no residual breast gland with tightened skin.^9^

### Nipple-areolar complex (NAC) plaster lifting technique

Another suggested technique uses an external plaster to lift and place excess skin in the desired position after liposuction. This technique was first used in a study^28^ with 30 participants, where 63% had Simon's grade IIb and the remaining 37% Simon's grade III. All patients underwent liposuction followed by excess glandular excision.

Lifting plasters were applied by lifting the NAC according to two different vectors of pull, enabling a redraping of the redundant skin and positioning the NAC at the desired position.

The plasters were held in place for 7 days for patients with Simon's grade IIb and two weeks for Simon's grade III. None of the participants had major complications; three patients developed seromas, which were treated conservatively. In five patients, the authors observed superficial skin necrosis at the periareolar incision site, which healed spontaneously after few weeks. No patient asked for surgical revision.

The same authors conducted a retrospective study of 448 patients.^29^ All of them benefited from UAL followed by glandular excision without skin excision. The NAC plaster lifting technique was applied to participants presenting gynecomastia grade IIb or III (37.7%). 116 patients experienced complications; seroma and superficial skin necrosis were the most common ones. Three cases of hematoma were also described. The follow-up time ranged from 6 to 14 months, and patients were asked to fill in a satisfaction questionnaire. 67.9% of patients were “very satisfied,” 25% “satisfied,” 7.1% “neutral, and 0% “not satisfied” with the general result. 92.9% reported a high level of satisfaction concerning their scars. Included patients presented with gynecomastia grade IIa (52.7%), followed by grade IIb (29.7%), grade III (8%), and grade I (4.7%). No data on grade-stratified patient-reported outcome were obtained.

## Discussion

Surgical correction of gynecomastia and lipomastia has been consistently reported to have a clear positive impact on patients.^6^ Many surgical techniques are described in the literature for the correction of gynecomastia, yet most of them leave stigmatizing scars when it comes to resection of the skin excess and are a common cause for dissatisfaction.^9^^,^^10^ The aim of this review was to summarize available skin-sparing techniques in the management of high-grade gynecomastia.

The use of LAL and UAL has found widespread resonance in general plastic surgery. In gynecomastia patients, they have been suggested to address light to moderate skin redundancy but have not yet found widespread acceptance for more severe skin excess.^30^

Some authors^26^^,^^31^^,^^32^ advocate that – if applied in the appropriate subdermal plane – UAL allows for skin contraction,^31^^,^^32^ avoiding the need of skin resection,^26^ and for minimization of bleeding in the postoperative period.^33^ However, no objective comparative data of skin retraction have been found in the literature. The authors suggest that skin retraction may be due to ultrasound's effects on the dermis and the subcutaneous fibrous septae.^34^ Tran et al. measured skin retraction rates following UAL between 15 and 35%, using permanent markers and sutures on the skin; but no control group was described.^35^

Concerning its mechanism of action, UAL is thought to have thermal, micro-mechanical, and cavitational effects on the surrounding tissues, causing cell destruction and fat liquefaction. Ultrasonic vibrations may apply mechanical pressure on fat cells, which is high enough to cause cell breakdown to liquid form, which is then absorbed by the body.^36^ The thermal effect spreads to the surrounding tissues, especially collagen, which is thought to be responsible for skin reshaping and tightening.^33^

Compared to classical liposuction, LAL is also thought to induce skin tightening and decrease bleeding.^37^ The laser–tissue interaction produces heat responsible for adipocyte membrane rupture, coagulation of small vessels, and collagen remodeling and thereby skin retraction.^37^ Trelles et al.^25^ advocate that collagen fibers, damaged by thermal energy during the procedure, need about 6–8 weeks to regenerate. However, skin tightening continues 6 months after treatment where the formation of new collagen fibers has been reported, which are then thought to be responsible for tissue reshaping and rejuvenation.^25^^,^^24^ If examined with an electron microscope, collagen fibers are seen to run parallel below the epidermal/dermal junction after LAL and thought to be responsible for tissue reshaping and to solve flaccidity.^38^ A decreased bleeding and postoperative incidence of hematoma, due to the thermal energy continually delivered during the treatment, was also described.^24^^,^^39^^,^^40^

RAL has found a large resonance in body contouring due to its advocated effect on skin retraction: a radiofrequency current is responsible for generating thermal energy acting directly on the tissues. This is thought to be responsible for thermal shrinking of the collagen fibers and liquefaction of adipose tissue, causing a reduced tissular volume and skin tightening.^41^ An *ex-vivo* study measured thermally induced collagen contraction in adipose tissue, dermis, and fascia by monitoring local temperature to define the shrinkage threshold.^42^ In volumetric histological analysis, soft tissue contraction has been described to reach between 31 and 47% six months postoperatively, which is significantly higher than that reported for other energy-emitting liposuction technologies.^27^^,^^42^

The combination of RAL and radiofrequency-assisted microneedling (Morpheus8 Body^Ⓡ^) is also used to reduce skin laxity.^43^ When using RAL only, dermal contraction has been reported to be weaker than the one in the subdermal plane, which could cause wrinkling of the overlying skin. The authors therefore advocate to combine surface microneedling to induce superficial skin tightening to smoothen the results.^10^

Independently of the surgical technique, patients’ characteristics play an important role in surgical outcomes. In fact, skin quality remains a key factor when it comes to expected postoperative skin retraction. It is known that skin quality varies for individuals or skin regions and that its constitution changes over time. In fact, Hodgson found a substantial difference in skin contraction depending on the age of the patient. ^34^ In cases of extreme skin excess, after massive weight loss, or in cases of poor skin quality, surgical skin resection most likely will remain the only viable option. However, we believe that a vast majority of patients, including those with Simon's grade III, are candidates for skin-sparing techniques as patients prefer having some residual skin laxity over stigmatizing scars. In line with that, Abdali et al. found in a randomized study, including patients with Simon's grade up to IIb, that participants who underwent liposuction only were more satisfied compared to the group undergoing liposuction with open excision. The fact of not having a stigmatizing scar emerged to be important for the participants.^44^ Another study retrospectively compared patient-reported satisfaction after gynecomastia surgery through a postoperative questionnaire. These were subdivided into three groups according to the type of operation: liposuction only, liposuction with excision, and open excision only. They found no statistically significant difference in overall satisfaction rates, but those who underwent liposuction only were more satisfied about the NAC appearance and the absence of NAC numbness and scars.^45^

### Our approach

Tumescent infiltration is performed with a solution of 1 L of 0.9% NaCl, 25 mL of 0.5% ropivacaine, and 1 mL of 1:100,000 epinephrine. Aggressive deep and superficial liposuction is performed on both breasts in a fanlike pattern using the Body-Jet^Ⓡ^ system (Human Med AG, Schwerin, Germany). An infra-areolar approach is used for glandular resection by paying attention to leave a 0.5 cm thick disk behind the NAC to avoid necrosis, nipple retraction, and areolar dimpling.

### Transcutaneous netting

**Figure 2:**
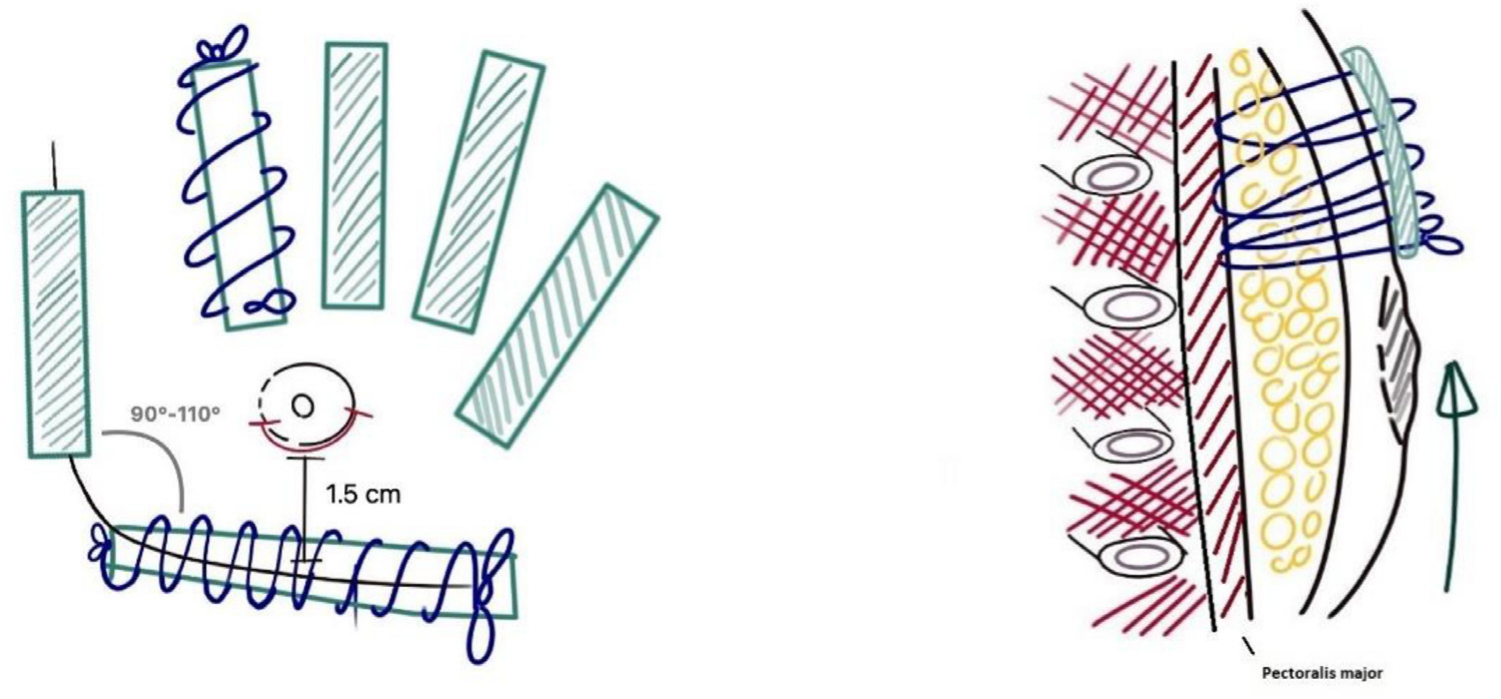
Schematic representation of our technique to address skin excess in coronal and sagittal view. Netting sutures (blue) involving subcutaneous tissue (yellow) and pectoral fascia (black) around sterile ophthalmic sticks (green).

The patient is put in a semi-sitting position. Depending on the skin excess, the NAC and periareolar skin are manually pulled in the desired position, i.e., 1.5 cm above the lower border of the pectoralis major muscle. To fix the skin in the desired position, transfixing continuous surgical sutures involving the skin and pectoralis fascia are performed using Prolene 2-0 around sterile ophthalmic sticks (Pro-Ophta, Lohmann & Rauscher AG, Switzerland) to avoid shearing on the skin. The sutures run in a radial fashion around the NAC and parallel to the lateral and inferior border of the pectoralis major muscle to promote definition of the latter. Care is taken to define a sharp angle (90–110°) between the inferior and lateral border of the pectoralis major muscle. This gives the breast a more square and masculine appearance (Figure 2).

**Figure 3:**
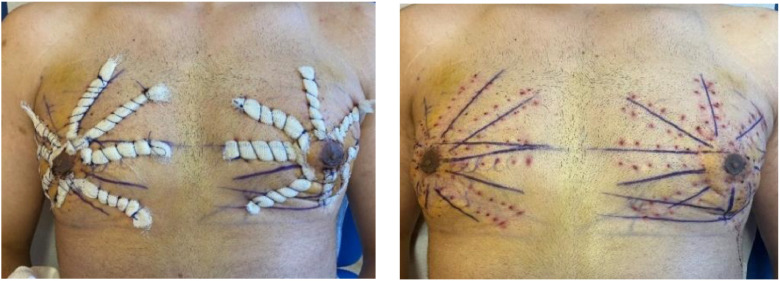
Picture at 5 days postoperative before and after removing netting sutures.

Transcutaneous netting not only allows for skin redraping, NAC positioning, and definition of the borders of the pectoralis muscle but also advocates a microneedling action on the skin (Figure 3).

**Figure 4:**
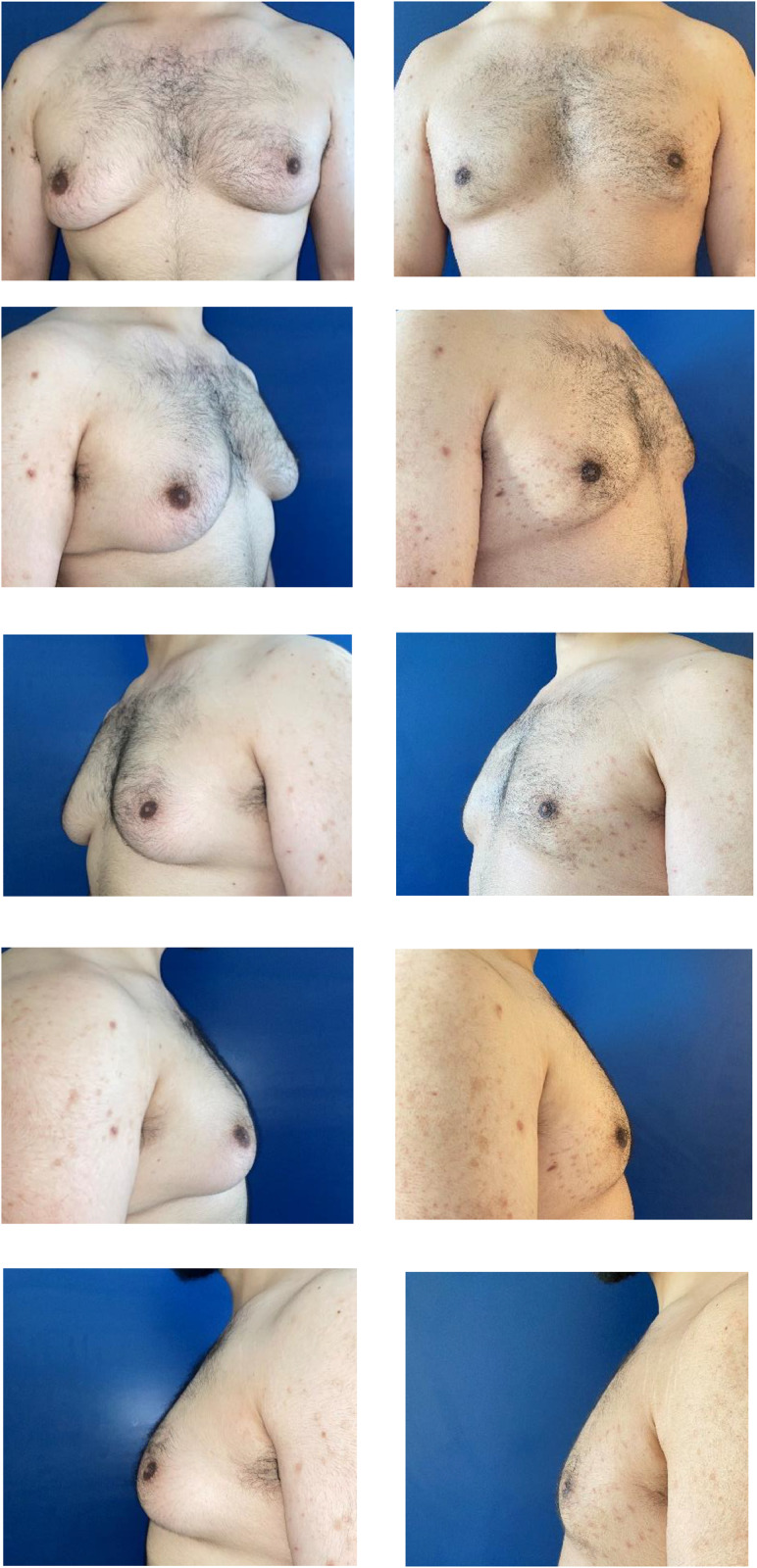
Preoperative (left) and 3 months postoperative (right) pictures of a patient presenting preoperatively gynecomastia grade III on the left and grade IIa on the right. Glandular resection was performed through a hemiareolar incision. Liposuction and transcutaneous netting were performed as described.

Finally, a compression bandage is applied. Patients are seen for follow-up at 24 and 48 h postoperatively, and the netting is removed at day 5 (Figure 4).

## Conclusions

Few data are available concerning skin-sparing techniques assessing postoperative skin excess in gynecomastia and lipomastia. Most of the literature consist of descriptive and anecdotal data, and we found very few comparative studies. Maximum follow-up was indicated at two^26^, three^34^, six,^24^^,^^25^ and fourteen months.^29^

Where skin redundancy is high, many authors still prefer skin resection, which is associated with potentially stigmatizing scars, which has been shown to play an important role in patients’ satisfaction.^44^ The available data do not allow for a conclusion on whether patients with high skin laxity have higher postoperative satisfaction rates when using skin-sparing techniques as no randomized controlled studies are available yet. The report that patients undergoing correction with liposuction only have a statistically higher satisfaction rate compared to those benefiting from liposuction combined with excision^44^ can be easily explained by the fact that patients who are classically chosen for skin-sparing techniques generally present with little skin excess and their results are inherently better. Advanced liposuction techniques, such as UAL, LAL, and RAL, hold promise to further push the limits of skin-sparing techniques in gynecomastia and lipomastia surgery, but controlled studies are lacking.

We advocate a skin-sparing technique for patients with gynecomastia, including Simon's grade III, using liposuction combined with transcutaneous netting, which allows for skin retraction and intraoperative fixation of the NAC at the desired position. Prospective studies assessing patient satisfaction rates after skin-sparing surgery with transcutaneous netting compared to skin resecting techniques are warranted.

## Ethical approval

Not required.

## Conflict of interest

One of the authors is the Editor-in-Chief for JPRAS Open and was not involved in the editorial review or the decision to publish this article. All remaining authors declare no conflict of interest
